# Elongated Hypocotyl 5-Homolog (HYH) Negatively Regulates Expression of the Ambient Temperature-Responsive MicroRNA Gene *MIR169*

**DOI:** 10.3389/fpls.2017.02087

**Published:** 2017-12-07

**Authors:** Phanu T. Serivichyaswat, Hendry Susila, Ji Hoon Ahn

**Affiliations:** Department of Life Sciences, Korea University, Seoul, South Korea

**Keywords:** miR169, HYH, G-box-like motif, ambient temperature, Arabidopsis

## Abstract

Arabidopsis microRNA169 (miR169) is an ambient temperature-responsive microRNA that plays an important role in stress responses and the floral transition. However, the transcription factors that regulate the expression of *MIR169* have remained unknown. In this study, we show that Elongated Hypocotyl 5-Homolog (HYH) directly binds to the promoter of *MIR169a* and negatively regulates its expression. Absolute quantification identified *MIR169a* as the major locus producing miR169. GUS reporter assays revealed that the deletion of a 498-bp fragment (–1,505 to –1,007, relative to the major transcriptional start site) of *MIR169a* abolished its ambient temperature-responsive expression. DNA-affinity chromatography followed by liquid chromatography-mass spectrometry analysis identified transcription factor HYH as a *trans-*acting factor that binds to the 498-bp promoter fragment of *pri-miR169a.* Electrophoretic mobility shift assays and chromatin immunoprecipitation–quantitative PCR demonstrated that the HYH.2 protein, a predominant isoform of HYH, directly associated with a G-box-like motif in the 498-bp fragment of *pri-miR169a*. Higher enrichment of HYH.2 protein on the promoter region of *MIR169a* was seen at 23°C, consistent with the presence of more HYH.2 protein in the cell at the temperature. Transcript levels of *pri-miR169a* increased in *hyh* mutants and decreased in transgenic plants overexpressing *HYH.* Consistent with the negative regulation of *MIR169a* by HYH, the diurnal levels of *HYH* mRNA and *pri-miR169a* showed opposite patterns. Taken together, our results suggest that HYH is a transcription factor that binds to a G-box-like motif in the *MIR169a* promoter and negatively regulates ambient temperature-responsive expression of *MIR169a* at higher temperatures in Arabidopsis.

## Introduction

Temperature is one of the major factors that govern plant development and physiological processes. For example, in *Arabidopsis thaliana* (Arabidopsis), exposure to a prolonged period of low temperature (i.e., vernalization) can shorten the time of flowering, especially in winter accessions. Small changes in temperature within the tolerable range, i.e., ambient temperature, also affect physiology and development of numerous plant species. For example, fluctuation of ambient temperature correlates with an increase of respiration rates (Atkin and Tjoelker, 2003), and higher ambient temperatures can shorten the time to flowering (Fitter and Fitter, 2002).

MicroRNAs (miRNAs) can induce sequence-specific gene silencing transcriptionally and post-transcriptionally (Voinnet, 2009), and regulate environmental responses, development, and hormone signaling in plants and animals (Bartel, 2009). In Arabidopsis, numerous miRNAs affect plant development. For example, miR169 affects plant responses to abiotic stress (Zhang et al., 2011a), cold stress (Zhou et al., 2008), salinity (Zhao et al., 2009), nitrogen deficiency (Zhao et al., 2011), and exposure to UV-B radiation (Zhou et al., 2007). MiR169 is also involved in development (Gonzalez-Ibeas et al., 2011) and floral initiation (Xu et al., 2014). Expression and accumulation of miR169 are significantly up-regulated at a low temperature (16°C), suggesting that miR169 may be involved in the ambient temperature response (Lee et al., 2010). Therefore, the transcriptional regulation of miR169 may be one of the key regulatory events in the ambient temperature response in plants.

MiR169 targets *Nuclear Factor Y Subunit A* (*NF-YA*) family genes (Jones-Rhoades and Bartel, 2004; German et al., 2008). NF-YA forms a heterotrimeric transcription factor complex with NF-YB and NF-YC subunits. The NF-Ys of animals are produced from single genes, but in plants, the three NF-Y subunits are encoded by multi-gene families (Edwards et al., 1998). The *A. thaliana* genome has 10 A subunit genes, 13 B subunit genes, and 13 C subunit genes (Siefers et al., 2009); this diversity could theoretically produce 1,690 unique NF-Y complexes. The NF-Y transcription factors act as positive transcriptional regulators but can also repress gene expression (Ceribelli et al., 2008; Leyva-Gonzalez et al., 2012).

Elongated Hypocotyl 5 (HY5) and its homolog HY5-Homolog (HYH) are basic leucine zipper (bZIP) transcription factors that function in lateral root formation, secondary root thickening, and photomorphogenesis of seedlings (Oyama et al., 1997). In *A. thaliana*, HYH is the closest homolog of HY5, showing 49% amino acid identity with HY5, especially in the DNA-binding basic domain (21 identical residues out of 24) and the bZIP transcription factor domain in the C-terminal half (Holm et al., 2002). Due to their high sequence similarity, HYH and HY5 bind to many of the same DNA motifs, including the G-box (Holm et al., 2002) and GATA box (Shi et al., 2011). In addition to their functions in transcriptional regulation in response to light, increasing evidence suggests that HYH and HY5 are involved in temperature acclimatization and transcriptional regulation (for instance, promotion of the transcription of *PEX11b*, *YUC8*, *XTR7*, and *EXP8*) (Hu and Desai, 2008; Zhang et al., 2011b,c; Toledo-Ortiz et al., 2014; Gangappa and Kumar, 2017; Park et al., 2017).

Although several reports have examined miR169-mediated regulation of target gene expression (Jones-Rhoades and Bartel, 2004; German et al., 2008), the upstream transcriptional regulation of *MIR169* itself remains unexplored. In this study, we identified *cis*-acting elements and a transcription factor involved in the regulation of ambient temperature-responsive expression of *MIR169*. We found that HYH binds the region of the *MIR169* promoter required for response to ambient temperature and represses *MIR169* expression. Taking our results together, we propose that HYH acts in the ambient temperature pathway as a transcription factor that binds to a G-box-like motif upon increasing temperature to repress the expression of *MIR169a.*

## Materials and Methods

### Plant Materials and Growth Conditions

*Arabidopsis thaliana* (L.) ecotypes Columbia (Col-0) and Wassilewskija-2 (Ws-2) were used in this study. The *hyh* mutants, which are in the Ws-2 background, were generously provided by Prof. Gareth Jenkins (University of Glasgow, United Kingdom). *MIM169*, a target mimic of miR169 that was designed to target miR169a, b, c, h, i, j, k, l, m, and n, was used to inhibit the miR169 activity (Todesco et al., 2010). Plants were grown in soil or on Murashige and Skoog (MS) media at 16 and 23°C under standard long-day (LD) conditions (16 h:8 h light:dark) with light intensity of 120 μmol m^-2^ s^-1^. Eight-day-old seedlings (unless otherwise indicated) were sampled and immediately frozen in liquid nitrogen prior to RNA or protein extraction.

### Generation of Transgenic Plants

For the promoter deletion analyses, the full-length promoter (2.1 kb) of *MIR169a* along with a series of 5′-deletions of the promoter (1.7, 1.2, 0.6, and 0.14 kb) were amplified from the genomic DNA using PCR. Each amplicon was named p169.1, p169.2, p169.3, p169.4, and p169.5, respectively. The full-length promoter and 5′-deletion fragments were then cloned into the pBI101 vector (Clontech, United States), which contains the β-*glucuronidase* (*GUS*) reporter gene. We named the resulting constructs *p169.1::GUS*, *p169.2::GUS*, *p169.3::GUS*, *p169.4::GUS*, and *p169.5::GUS*. To generate *MIR169a-*overexpressing plants, the genomic fragment of *MIR169a* was amplified by PCR, and then cloned into the pCHF3 binary vector harboring the 35S promoter. To generate *HYH-*overexpressing plants, *HYH* cDNAs were amplified from total RNA using RT-PCR and then cloned into the pBA-HA binary vector. The sequence of each plasmid construct was verified by sequencing (Cosmogenetech, South Korea). The constructed plasmids were then introduced into wild-type Col-0 plants using the floral dip method (Clough and Bent, 1998). The homozygous transgenic plants were isolated and used for the subsequent experiments. Information on the primers that were used in this study is shown in Supplementary Table 1.

### Gene Expression Analyses

Total RNA was extracted from 8-day-old seedlings using Plant RNA Purification Reagent (Invitrogen, United States). The extracted RNA was treated with DNase I (NEB, United States) to eliminate DNA contamination, then cDNA was synthesized using the Transcriptor First-Strand cDNA Synthesis Kit (Roche Applied Science, United States). The transcript levels were measured by quantitative real-time PCR (qRT-PCR), using Green I Master Mix (Roche Applied Science, United States) with specific primers (Supplementary Table 1). The data were normalized against two stable reference genes, *PP2AA3* (*AT1G13320*) and a *SAND* family gene (*AT2G28390*) (Hong et al., 2010). All reactions were carried out with three biological replicates, each with three technical replicates. The absolute quantification was performed as previously described (Whelan et al., 2003), using serial dilutions of known concentrations of the cloned open-reading frames for the generation of the standard curve. For the analyses of the expression of *HYH* splice variants, our published RNA sequencing (RNA-seq) data for Col-0 plants grown at 23°C (GSE87851) (Nasim et al., 2017) were downloaded and analyzed.

For western blot experiments, total proteins were extracted with Pro-Prep protein extraction buffer (Intron Biotechnology) using 8-day-old seedlings grown on MS media supplemented with 1.5% sucrose at 16 and 23°C under LD conditions. Samples were harvested at zeitgeber time 4 (ZT4). HA:HYH protein was detected using monoclonal anti-HA-antibodies (Sigma, H9658) and polyclonal anti-Actin-antibodies (Agrisera) were used to detect actin, which was used as a loading control.

### Histochemical and Fluorometric GUS Assays

Histochemical localization of GUS was analyzed in the 8-day-old transgenic seedlings expressing *GUS* under the control of the *MIR169a* promoter fragments. The samples were incubated for 12 h at 37°C with the substrate solution (1 mM 5-bromo-4-chloro-3-indolyl-β-D-glucuronide, pH 7.0, 100 mM sodium phosphate buffer, 10 mM Na_2_EDTA, 0.5 mM potassium ferricyanide, 0.5 mM potassium ferrocyanide, and 0.1% Triton X-100). Stained seedlings were washed with 70% ethanol to eliminate chlorophyll, and were then photographed with a Nikon SMZ1000 Stereomicroscope (Tokyo, Japan). GUS enzymatic activity was quantified using the GUS Fluorescent Reporter Gene Activity Detection Kit (Sigma, United States) according to the manufacturer’s instructions. The assay was performed with three biological replicates, each with three technical replicates. Statistical analysis for the enzymatic activity was conducted using one-way analysis of variance (ANOVA), and the means were compared using Duncan’s Multiple Range Test.

### DNA-Affinity Chromatography and Liquid Chromatography-Mass Spectrometry (LC-MS)

DNA-binding proteins were enriched using DNA-affinity chromatography as previously described (Jutras et al., 2012). Total protein was extracted from 8-day-old Col-0 seedlings grown on MS media at 16 and 23°C. A bait DNA was designed based on the 498-bp fragment (-1,505 to -1,007, relative to the major transcription start site, TSS) on the *MIR169a* promoter, and generated from the genomic DNA using PCR with one 5′-biotin-modified and one unmodified primers (Supplementary Table 1). The biotin-labeled bait DNA was affixed to Dynabeads MyOne Streptavidin T1 (Invitrogen, United States). Elution was carried out with various NaCl concentrations. The eluents were then subjected to SDS gel electrophoresis, and visualized by silver staining. A single band that was eluted in the 200 mM NaCl buffer was excised, and subjected to Liquid Chromatography-Mass Spectrometry (LC-MS) (Korea Basic Science Institute). Peptide sequences were compared against Arabidopsis proteins using Protein BLAST^1^. Significant parameters were fixed at *p* < 0.05.

### Recombinant Protein Production and Purification

To produce His-tagged HYH protein, the open-reading frame of Arabidopsis *HYH.2* (the major splice variant of *HYH*) was cloned from cDNA using RT-PCR (primer information in Supplementary Table 1), and ligated into the pET28a+ vector (EMD Biosciences, United States). The cloned insert was sequenced to confirm that it lacked mutations, was in-frame, and had the histidine tag. The plasmid was then introduced into *Escherichia coli* BL21 cells. The recombinant protein was induced by the addition of 1 mM IPTG after 3 h of incubation at 37°C in LB media. The IPTG-induced cell pellets were collected by centrifugation, resuspended in 1× His-binding buffer (5 mM imidazole, 0.5 M NaCl, 20 mM Tris–HCl pH 7.9), sonicated, then centrifuged. The supernatant containing soluble HYH.2-His protein was collected and then purified using His GraviTrap (GE Healthcare Life Sciences, United States). SDS–PAGE and western blotting using anti-His antibody were then carried out to check the protein quality and identity, respectively.

### Electrophoretic Mobility Shift Assays (EMSAs)

The DNA sequences of the predicted HYH-binding motifs were synthesized with a 5′-biotin label (Cosmogen, Korea). Electrophoretic mobility shift assays (EMSAs) were performed using the LightShift Chemiluminescent EMSA Kit (Thermo Scientific, United States) according to the manufacturer’s instructions. Double-stranded 5′-biotin-labeled oligonucleotides were used as DNA probes (Supplementary Table 1), and together with the purified HYH.2-His protein (100 ng), the reactions were incubated at room temperature for 30 min. The reaction samples were then subjected to electrophoresis in an 8% native polyacrylamide gel and transferred to a nylon membrane. Visualization was performed as described in the manufacturer’s instructions (Thermo Scientific, United States).

### Chromatin Immunoprecipitation (ChIP)–qPCR

Chromatin immunoprecipitation (ChIP) was performed as described (Song et al., 2016) using 12-day-old seedlings grown on MS media supplemented with 1.5% sucrose at 16 and 23°C under LD conditions. Samples were harvested at ZT4. The genomic fragment was immunoprecipitated with monoclonal anti-HA-antibodies (Sigma, H9658) conjugated with Dynabeads Protein G (Thermo Scientific, 10003D). The relative enrichment of each amplified fragment was analyzed according to the “% of input” method (Haring et al., 2007). ChIP assay was carried out in two biological replicates, each with three technical replicates. qPCR was done using the primers shown in Supplementary Table 1.

## Results

### *MIR169a* Is the Major Locus of the miR169 Family

**FIGURE 1:**
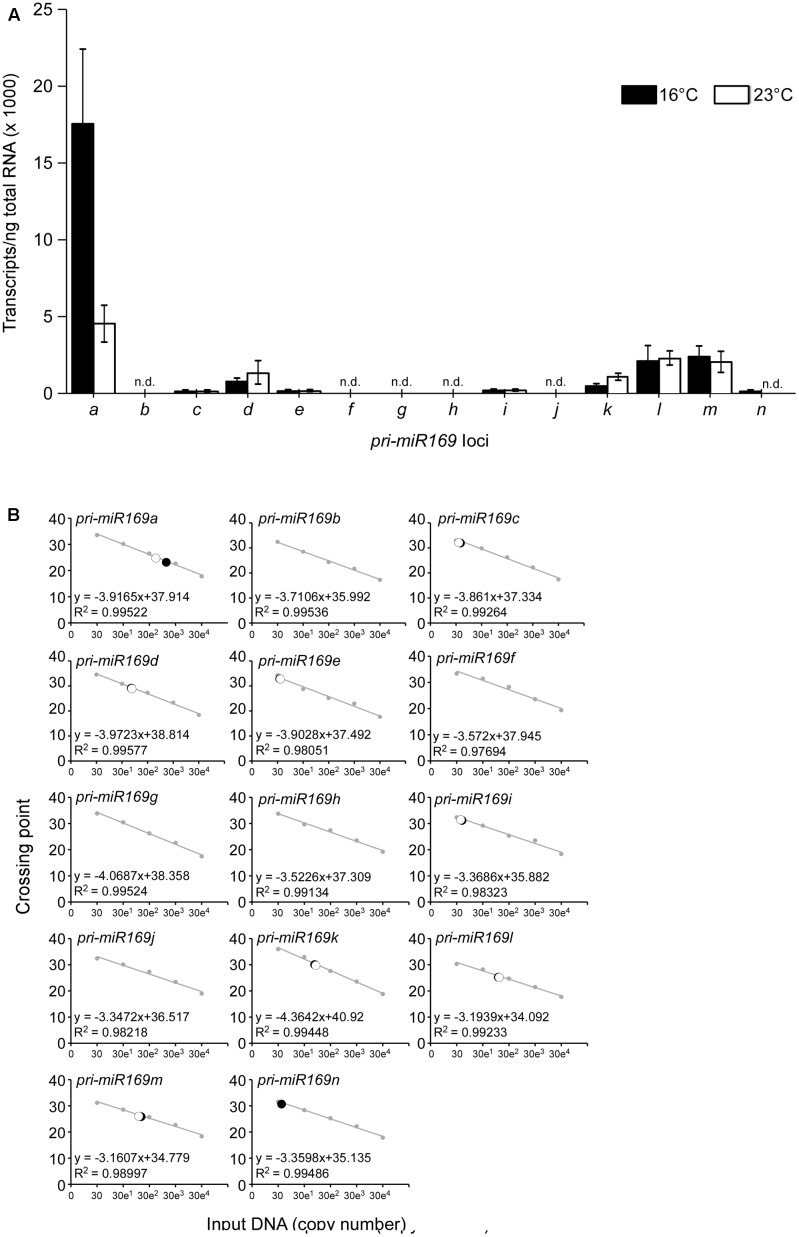
Absolute quantification of *pri-miR169* species at 23 and 16°C. **(A)** Absolute quantification of the abundance of the primary transcripts of each *MIR169* in 8-day-old Col-0 grown at 23 and 16°C under LD conditions. All values are presented as the mean ± SD of three biological replicates. n.d., not detectable. **(B)** The regression line from the standard curves used to determine the concentration of each *pri-miR169* transcript at 23 and 16°C. The set of standards (gray) contains 10-fold serial dilutions from 30 to 300,000 copies of each transcript. The regression line from the dilution curve was used to determine the concentration of each transcript. Closed circles indicate data points at 16°C and open circles indicate data points at 23°C. Crossing point represents Ct-values from the qPCR.

Mature miR169 is generated from 14 loci in the *A. thaliana* genome, comprising the largest family among Arabidopsis miRNAs. To identify the major locus of the miR169 family, we performed absolute quantification using qPCR to determine the abundance of transcripts from each *pri-miR169* locus from wild-type Arabidopsis plants at 23 and 16°C. As shown in **Figure 1**, *pri-miR169a* was the most abundant of all transcripts generated from the loci (∼20,000 transcripts/ng of total RNA at 16°C and ∼5,000 transcripts/ng of total RNA at 23°C), followed by *pri-miR169m*, *l*, *k*, and *d*, which had similar amounts of transcripts (∼1,000–3,000 transcripts/ng of total RNA). By contrast, *pri-miR169g*, *f*, *c*, *i*, *b*, and *j* showed very low levels of transcripts (<500 transcripts/ng of total RNA at both temperatures). The results suggested that *MIR169a* was the major locus of the miR169 family at both 23 and 16°C and furthermore its expression levels were approximately four times higher at 16 than at 23°C. The 14 loci produce four mature miR169 isoforms; therefore, we suggest that the miR169a isoform has the highest abundance, followed by miR169h–n, d–g, and bc. Consistent with our observation, deep-sequencing results also suggest that *MIR169a* is the major locus of the miR169 family (Bologna et al., 2013), although levels of the minor isoforms are partly inconsistent with our results, most likely due to the different quantification methods used. Based on its high level of expression, we selected *MIR169a* for subsequent experiments in this study.

### The *MIR169a* Promoter Contains Several Regulatory Elements That Are Involved in Environmental Responses

**FIGURE 2:**
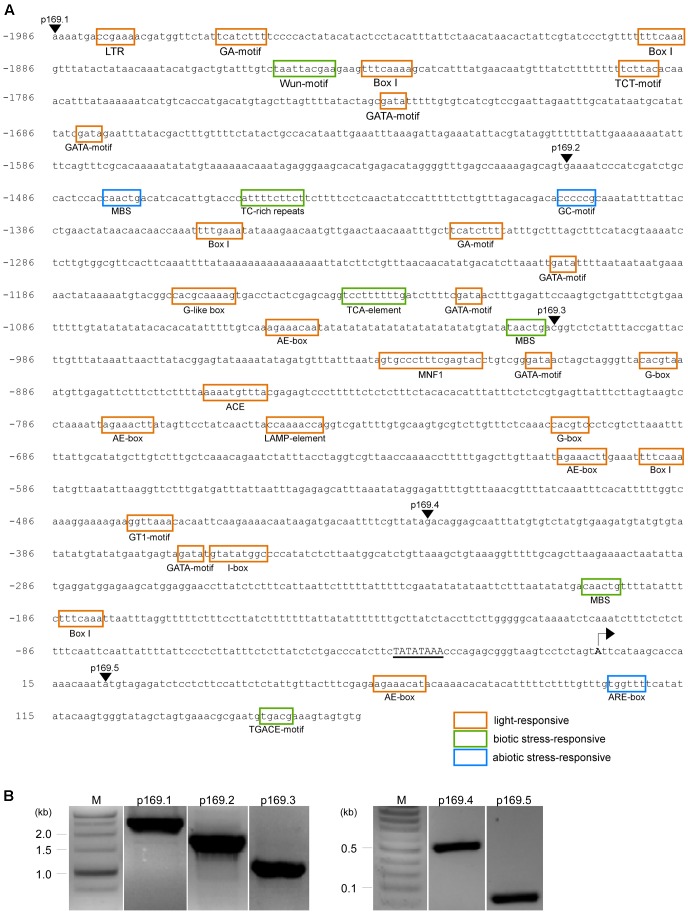
*In silico* analysis and cloning of the *MIR169a* promoter. **(A)** Putative *cis*-acting elements identified in the full-length promoter (2,149 bp) of *MIR169a* by PlantCARE (Lescot et al., 2002). The putative *cis*-acting elements involved in light signaling, biotic stresses, and abiotic stresses are indicated by orange, green, and blue boxes, respectively. The arrowheads indicate the location of the forward primers used for the amplification of the full-length promoter (*p169.1*) and its 5′-deletion derivatives (*p169.2* to *p169.5*). A putative TATA box is underlined. The reported major TSS (Xie et al., 2005) is indicated with an arrow. The location is relative to the TSS. **(B)** Amplification of the full-length promoter of *MIR169a* (*p169.1*) and its 5′-deletion derivatives (*p169.2* to *p169.5*). M, molecular marker.

To initially narrow down the important DNA regions that are responsible for the ambient temperature responsiveness of the *MIR169a* promoter, we performed a 5′-promoter deletion analysis. We cloned approximately 2 kb of the genomic region upstream from the previously reported major TSS of *MIR169a* (Xie et al., 2005). Analysis of the *cis*-acting elements of the *MIR169a* promoter using PlantCARE^2^ showed the presence of 19 different classes of potential *cis*-acting elements that may be involved in environmental responses (**Figure 2A** and Supplementary Table 1). These included 11 light-responsive elements (i.e., GA-motif, Box I, TCT-motif, GATA-motif, AE-box, MNF1, G-box, ACE, LAMP-element, GT1, and I-box), four abiotic stress-inducible elements including a low temperature-responsive (LTR) element, a drought-inducible element (MBS), an enhancer-like element involved in anoxic-specific inducibility (GC-motif), and an anaerobic induction element (ARE-box), and four defense-responsive elements including a wound-responsive element (Wun-box), a defense and stress-responsive element (TC-rich repeats), a salicylic acid-responsive element (TCA-element), and a jasmonate-responsive element (TGACG). The identification of these putative *cis*-acting elements suggests that the promoter of *MIR169a* may respond to various environmental signals, including low temperature, light, drought, anoxia, and pest and pathogen attack.

### Generation of Transgenic Plants Containing the *MIR169a* Promoter Deletion Constructs

The full-length promoter (2,149 bp) of *MIR169a* was amplified from the genomic region upstream of the first *MIR169a* intron (Lane p169.1, **Figure 2B**) by PCR and then cloned into the promoter-less binary vector pBI101 containing a *GUS* reporter gene, and named *p169.1::GUS*. In addition, promoter deletion fragments with serial deletions of approximately 500 bp from the 5′-end were generated using PCR, which resulted in promoter fragments of 1,668, 1,170, 590, and 141 bp (primer information in Supplementary Table 1) (**Figure 2B**). These fragments were cloned into the promoter-less binary vector pBI101 to generate *p169.2::GUS*, *p169.3::GUS*, *p169.4::GUS*, and *p169.5::GUS* constructs, respectively. All constructs were introduced into wild-type Arabidopsis Col-0 using the *Agrobacterium*-mediated floral dip method. The transformants were screened on MS media containing kanamycin, and then checked for the presence of the transgene insertion by PCR (data not shown). Homozygous lines were isolated from the T_3_ generation and used for subsequent experiments.

### Promoter Deletion Analyses Identified a 498-bp Region Required for Ambient-Temperature Responsiveness

To examine the ambient temperature inducibility and identify the ambient temperature-responsive region of the *MIR169a* promoter, we first performed fluorometric GUS assays using transgenic plants containing the different *MIR169a* promoter fragments. At 23°C, GUS activities of plants harboring *p169.1*, *p169.2*, and *p169.3* constructs were high but their levels were not statistically different. However, at 16°C, the highest levels of GUS activity were observed in the transgenic plants harboring *p169.1* and *p169.2* construct, and the activity was significantly induced at 16°C by approximately 2.7-fold, consistent with a previous study that reported higher accumulation of mature miR169 at 16°C (Lee et al., 2010). Since the *GUS* mRNA is not processed by the miRNA biogenesis machinery, these results suggested that the expression levels of *MIR169a* are proportional to the amount of the mature miR169a and the effects of ambient temperature on *MIR169a* occur at the transcriptional level. The induction at 16°C was slightly reduced in *p169.2::GUS* plants, but still approximately 2-fold higher than at 23°C. However, a significant loss of GUS activity at 16°C was observed in *p169.3::GUS* plants, resulting in similar GUS activities at 23 and 16°C. These results suggested that the 498-bp promoter region (-1,505 to -1,007, relative to the major TSS) was crucial for the ambient temperature response, raising a possibility that the regulatory elements are present in the 498-bp fragment. Further deletion of promoter to the -427 position (*p169.4::GUS*) caused a dramatic loss of promoter activity at both temperatures, indicating that the core promoter of the *MIR169a* gene is located between the -1,007 and -427 positions. GUS activity was completely abolished in the +22 deletion (*p169.5::GUS*). The *pBI101* transgenic line, which contains a promoter-less *GUS* gene, showed no induction at both temperatures, as seen in *p169.5::GUS* plants.

**FIGURE 3:**
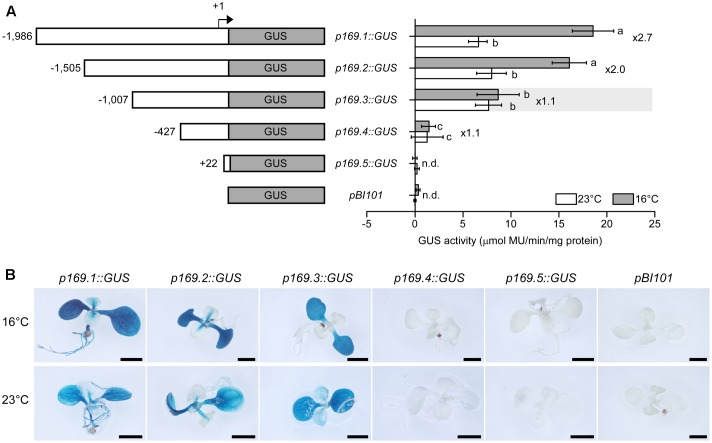
Deletion analysis of the *MIR169a* promoter. **(A)** Fluorometric GUS assays using transgenic plants expressing *GUS* under the control of different *MIR169a* promoter fragments at 23 and 16°C. Diagrams of the constructs used for GUS expression assays are shown on the left. Numbers in the diagram indicate the 5′-end points of the promoter fragments relative to the TSS (denoted as +1). The transgenic plants harboring *GUS* lacking a promoter (*pBI101*) were used as a negative control. The columns that are significantly different based on Duncan’s Multiple Range Tests (*p* < 0.05) are marked with different letters. The ratio of the promoter activity at 16°C to that at 23°C, i.e., the induction ratio, is indicated on the right. The values are presented as the mean ± SD of three biological replicates. n.d., not detectable. **(B)** Histochemical assay for GUS activity and tissue-specific localization. Transgenic plants harboring each construct were grown at 16 and 23°C. At least 10 seedlings were examined and typical results are presented. Scale bar = 2 mm.

To investigate the spatial and tissue-specificity of *MIR169a* promoter activity, the homozygous *pMIR169::GUS* plants were subjected to histochemical GUS staining. As expected, strong GUS staining was observed in *p169.1::GUS* plants at 16°C. Strong staining was detected in the cotyledons of the seedlings grown at 16°C. The hypocotyl, root, and true leaves also showed weaker GUS staining. Similar staining patterns were observed at 23°C but to a comparatively lesser extent (**Figure 3B**). In *p169.2::GUS* seedlings, the staining was still strongly detected in the cotyledon and hypocotyl, but not in the root. This implies that the region between -1,986 and -1,505 of the promoter contains a sequence that confers root-specific expression. The overall GUS staining intensity was higher at 16°C than at 23°C in *p169.2::GUS* plants, consistent with the results from the enzymatic activity assays. Notably, no apparent difference in GUS staining intensity was observed in *p169.3::GUS* plants at the two different temperatures. The *p169.4::GUS* and *p169.5::GUS* plants showed no visible GUS staining. The *pBI101* plants, which contain the promoter-less *GUS* gene, did not show any GUS staining. These results suggested that the expression of *MIR169a* is upregulated by a low ambient temperature at the transcriptional levels and a *cis*-acting element that responds to ambient temperature changes is located in the 498-bp fragment (-1,505 to -1,007) of the *MIR169a* promoter.

### The HYH Transcription Factor Binds to the 498-bp Fragment of the *MIR169a* Promoter

**FIGURE 4:**
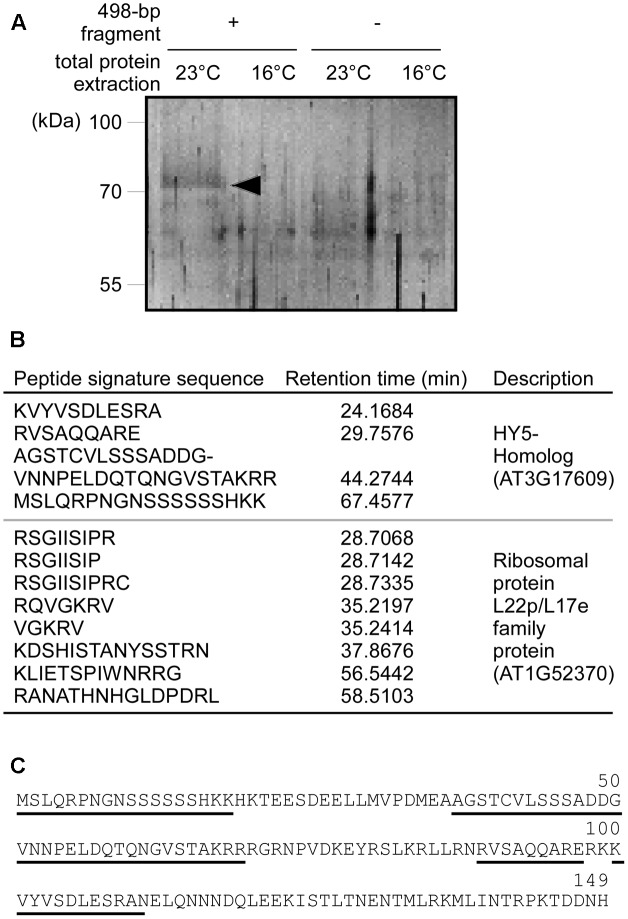
DNA-affinity chromatography and identification of HYH as a protein that binds to the 498-bp fragment of the *MIR169a* promoter. **(A)** SDS–PAGE following DNA-affinity chromatography. Total protein extracts were obtained from wild-type plants grown at 23 and 16°C, and purified by affinity chromatography using the 498-bp fragment as a DNA bait. Note that an apparent band (arrowhead) appeared in the first lane (498-bp fragment bait + total protein extracted at 23°C). **(B)** Peptide signature sequences of the band in **(A)**, obtained by LC-MS. The retention time of each peptide is shown in the left column. **(C)** Amino acid sequence of Arabidopsis HYH protein. The sequences of the recovered peptides from LC-MS **(B)** are underlined.

The deletion analysis showed that the 498-bp fragment is important for ambient temperature-responsive *MIR169a* expression; we therefore postulated that transcription factors involved in the ambient temperature response might bind to this region and regulate *MIR169a* expression. To identify such transcription factor(s), DNA-affinity chromatography was performed, using the 498-bp fragment as a DNA bait, and total protein extracted from 8-day-old seedlings grown at 16 and 23°C. We eluted affinity-captured proteins and visualized them after SDS-PAGE. After silver staining, we identified a single band (about 75 kDa) at 23°C (arrowhead in **Figure 4A**), but not at 16°C, suggesting that the eluted proteins bind to the 498-bp fragment of the *MIR169a* promoter *in planta*. The control reactions that used the same protein extracts without the 498-bp fragment as a bait did not elute these proteins.

To identify the unknown proteins, the band in **Figure 4A** was excised from the gel and then subjected to LC-MS to identify its amino acid composition. Twelve peptide signature sequences were recovered by LC-MS. By comparing them against the Arabidopsis protein database using Protein BLAST, four peptide sequences were identified as being encoded by the ORF of *AT3G17609*, which encodes the bZIP transcription factor protein Elongated Hypocotyl 5-Homolog (HYH) (**Figures 4B,C**). The remaining eight peptide sequences belonged to the ribosomal protein L22p/L17e family protein encoded by *AT1G52370* (**Figure 4B**). As the ribosomal protein is thought to be ubiquitous in the cytosol, we regarded the ribosomal protein as a contaminant. These results suggest that HYH protein directly interacts with the *cis*-acting element(s) that are present within the 498-bp fragment (-1,505 to -1,007, relative to the major TSS) of the *MIR169a* promoter to regulate *MIR169a* expression at 23°C.

### HYH.2 Is the Most Abundant Isoform among the Splice Variants of *HYH*

**FIGURE 5:**
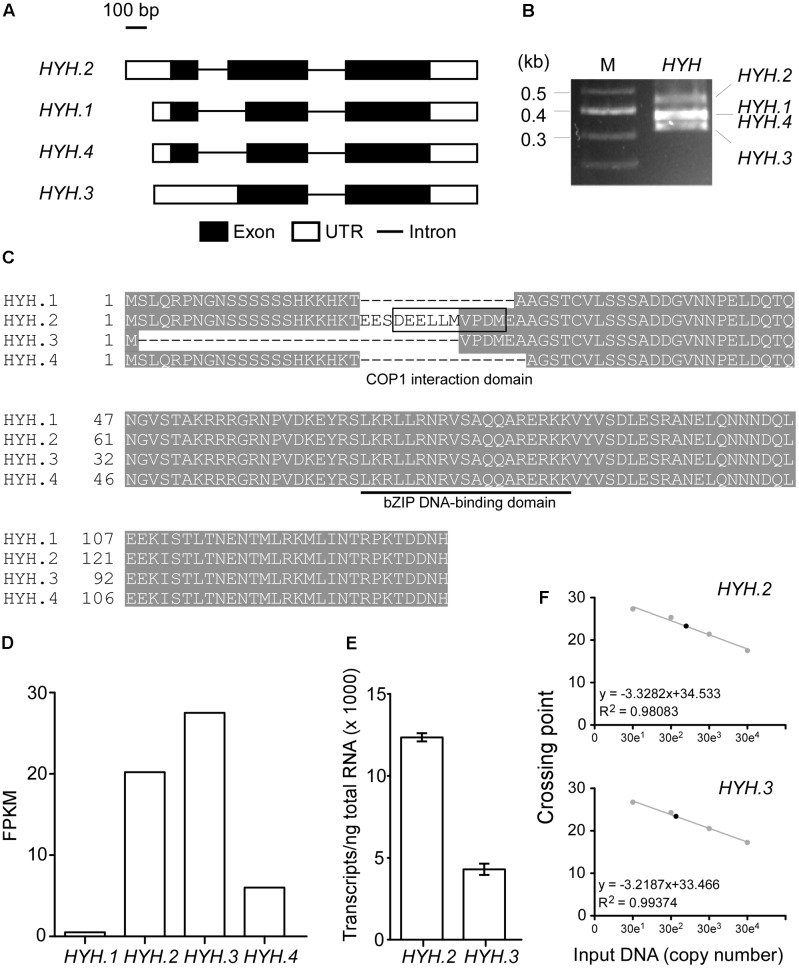
Identification of splicing variants of *HYH* and determination of its major isoform. **(A)** Schematic diagrams showing the four splicing variants of *HYH*. **(B)** Amplification of splicing variants of *HYH* using RT-PCR. M, molecular marker. **(C)** Multiple amino acid sequence alignment of four HYH protein isoforms. The COP1-binding domain is indicated by a box, and the bZIP DNA-binding domain is underlined. **(D)** RNA-seq-quantitative measurement of four *HYH* isoforms. FPKM, fragments per kilobase of transcript per million mapped reads. **(E)** Absolute quantification of the abundance of mRNAs of *HYH.2* and *HYH.3*. The values are presented as the mean ± SD of three biological replicates. **(F)** The regression line from the standard curves used to determine the concentration of *HYH.2* and *HYH.3* transcripts. The set of standard curves contains 10-fold serial dilutions from 300 to 300,000 copies of each transcript. Closed circles indicate data points. Crossing point represents Ct-values from the qPCR.

The recently released Araport version 11 revealed that nearly 40% of protein-coding loci produce two or more splicing isoforms (Cheng et al., 2017) and alternative splicing plays an important role in ambient temperature responses (Lee et al., 2013; Pose et al., 2013). Therefore, we analyzed our published RNA-seq data to find splicing variants of *HYH* (Nasim et al., 2017). The analysis revealed that four splicing variants of *HYH* are present in wild-type plants (**Figure 5A**), consistent with a previous report (Li et al., 2017). To confirm the alternative spliced isoforms, we performed RT-PCR using RNA isolated from wild-type plants. Although three amplified bands were visible (**Figure 5B**), we identified four splicing variants (*HYH.1*–*HYH.4*) after sequencing. This was due to the fact that amplicons from *HYH.1* and *HYH.4* were only 3-nt different in size (i.e., 408 and 405 bp, respectively). Amino acid alignment showed that of the four variants, *HYH.2* is the longest, followed by *HYH.1*, which contains an extra codon at the 3′-splicing site, compared to *HYH.4* (**Figure 5C**). *HYH.3* appeared to be the shortest. Although all four HYH isoforms harbor the bZIP DNA-binding domain (underlined in **Figure 5C**), only HYH.2 contains the Constitutive Photomorphogenic1 (COP1) interaction domain, which was previously suggested to have a non-canonical interaction with COP1 (Li et al., 2017).

Our RNA-seq data (Nasim et al., 2017) suggested that *HYH.2* and *HYH.3* had similarly high transcript levels [20.2 and 27.5 fragments per kilobase of transcript per million mapped reads (FPKM), respectively] (**Figure 5D**). To validate the RNA-seq results, absolute quantification was performed to quantify and compare the transcript abundance of *HYH.2* and *HYH.3* splicing variants in 8-day-old wild-type plants at 23°C. The results showed that *HYH.2* had ∼13,000 transcripts/ng of total RNA, while *HYH.3* had ∼4,000 transcripts/ng of total RNA (**Figures 5E,F**), implying that *HYH.2* is the most abundant *HYH* splicing variant. Therefore, we chose *HYH.2* for the subsequent experiments.

### HYH Binds to a G-Box-Like Motif within the *MIR169a* Promoter

**FIGURE 6:**
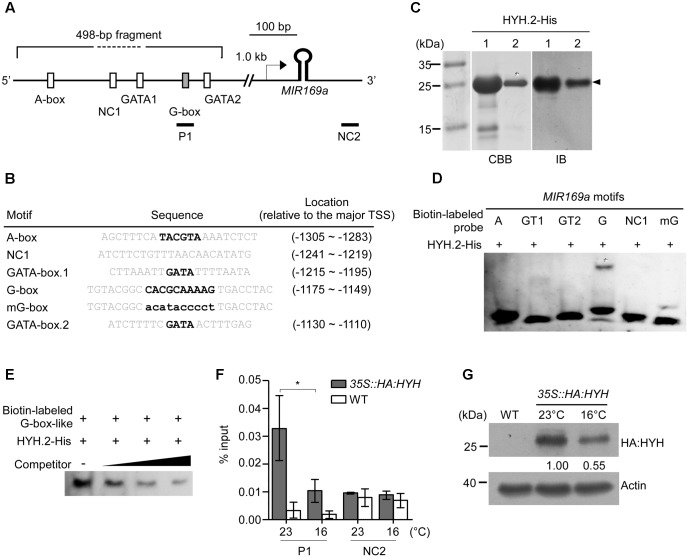
Electrophoretic mobility shift assays (EMSAs) and ChIP–qPCR analyses showing that HYH binds to a derivative of the G-box motif. **(A)** Schematic representation of the locations of four putative HYH-binding motifs within a 498-bp fragment of the *MIR169a* promoter. The reported major TSS (Xie et al., 2005) is denoted with an arrow. P1: a region (–1,353 to –1,273, relative to the major TSS) containing the G-box-like motif that was used for amplification in the ChIP–qPCR assay. NC1, negative control for the EMSAs, NC2, negative control for the ChIP–qPCR assays (+485 to +604, relative to the major TSS). **(B)** Sequence information for the putative motifs used as probes for the EMSA. Core motifs are marked in bold. **(C)** Production and purification of His-tagged HYH.2 proteins. SDS gel electrophoresis of column-purified recombinant HYH.2 protein (lanes 1 and 2, middle panel). The arrowhead indicates the soluble HYH.2-His protein of the expected size. Immunoblotting was performed using anti-His antibodies to confirm the purity of the HYH.2-His proteins (arrowhead, right panel). The eluted protein from lane 2 (asterisk) was subsequently used for further experiments. CBB, Coomassie brilliant blue staining; IB, immunoblot. **(D)** EMSA with HYH.2-His protein. Shifted bands are indicated with an asterisk. A, A-box, GT1; 2, GATA box 1 and 2; G, G-box-like motif; mG, mutant G-box-like motif; NC1, negative control. **(E)** Competition assay of HYH.2-His protein binding to the G-box-like motif. Unlabeled probes of identical sequence were used as competitors at 100×, 400×, and 1,600× molar excess. Only shifted bands are shown for simplicity. **(F)** ChIP–qPCR analyses to determine enrichment of HYH protein on the *MIR169a* promoter. Chromatin of *35S::HA:HYH* and WT plants grown at 16 and 23°C was immunoprecipitated with anti-HA antibody. ChIP results are presented as the percentage recovered from the total input DNA (% input). All values are presented as the mean ± SD of three technical replicates, ^∗^*p* ≤ 0.05 (Student’s *t*-test). **(G)** Determination of HYH protein levels at 23 and 16°C. Total proteins of *35S::HA:HYH* grown at 23 and 16°C were immunoblotted with anti-HA antibody. Actin was used as a loading control. Bands were quantified using Image J software and the numbers below each band denote fold change relative to the HA:HYH level at 23°C.

To identify the DNA motif(s) that were bound by HYH, we conducted *in silico* analyses, which identified four potential HYH-binding motifs within the 498-bp fragment of the *MIR169a* promoter: one A-box (Izawa et al., 1993), two GATA-boxes (Shi et al., 2011), and one derivative of the G-box (here denoted as G-box-like) (Holm et al., 2002) (**Figure 6A**). The sequences of the identified motifs and their locations relative to the major TSS (Xie et al., 2005) are shown in **Figure 6B**.

To investigate whether HYH binds to the *cis*-acting elements(s) in the promoter of *MIR169a*, recombinant His-tagged full-length soluble HYH.2 protein (approximately 20 kDa) was produced and purified on histidine affinity columns. The purified protein displayed a single major band after elution from the column at the expected molecular weight (asterisks in **Figure 6C**). We performed an immunoblot analysis using anti-His antibody to confirm that the purified product contained HYH.2-His protein. Anti-His antibody successfully detected HYH.2-His protein at the expected size after blotting (**Figure 6C** right panel).

To confirm that HYH protein indeed bound to the DNA motif(s) and was responsible for the interaction with the promoter, an EMSA was performed. Purified HYH.2-His protein and synthesized double-stranded 5′-biotin-labeled oligonucleotides of the possible HYH-binding motifs (**Figure 6B**) were allowed to interact before being separated by electrophoresis. The results showed that HYH.2-His bound to the G-box-like motif, as a shifted band was observed for the G-box-like DNA probe. This suggested that HYH binds to the G-box-like motif within the 498-bp fragment of the promoter of *MIR169a*. In contrast, the A-box, both GATA boxes, as well as the negative control (NC1) did not show detectable shifted bands (**Figure 6D**). To test whether the G-box-like sequence was accountable for the interaction, the core of the motif was mutated with G to T, or A to C (denoted as mG, **Figure 6B**). The binding reaction between HYH.2-His and mG failed to produce any apparent band-shifts (**Figure 6D**), suggesting that HYH specifically binds to the G-box-like motif in the 498-bp fragment. To further confirm the binding affinity, unlabeled competitor probe of the identical sequence was used to perform the competition assay. The results showed that the unlabeled probe was able to compete for HYH protein binding to the G-box-like motif, as the intensity of the shifted bands gradually decreased with the increasing concentrations of unlabeled probe (**Figure 6E**).

To further confirm the association of HYH with the *MIR169a* promoter region *in vivo*, we performed ChIP–qPCR assays. Chromatin from *35S::HA:HYH* plants grown at 16 and 23°C was immunoprecipitated with anti-HA antibody and the immunoprecipitated DNA was used as a template for amplification of the *MIR169a* promoter region covering the G-box-like motif (P1). A region downstream of the hairpin loop structure of miR169 was also used as a negative control (NC2) for ChIP–qPCR experiments (**Figure 6A** and Supplementary Table 1). The results showed apparent binding of HYH in the P1 region of the *MIR169a* promoter at 23°C, whereas less enrichment of HYH in the P1 region was observed in the plants grown at 16°C. In contrast, we observed no enrichment in the NC2 region (**Figure 6F** and Supplementary Figure 1A). We then examined HYH protein levels using *35S:HA:HYH* plants grown at 23 and 16°C. An immunoblot analysis showed that HA:HYH protein levels were higher at 23°C than at 16°C (**Figure 6G** and Supplementary Figure 1B), consistent with higher enrichment of HYH protein in the P1 region of the *MIR169a* promoter (**Figure 6F**). Taken together, our results suggested that HYH protein directly binds to the G-box-like motif of *MIR169a in vitro* and *in vivo*, especially at 23°C, to regulate its transcription.

### *HYH* Acts Upstream of *MIR169a* to Negatively Regulate *MIR169a* Expression

**FIGURE 7:**
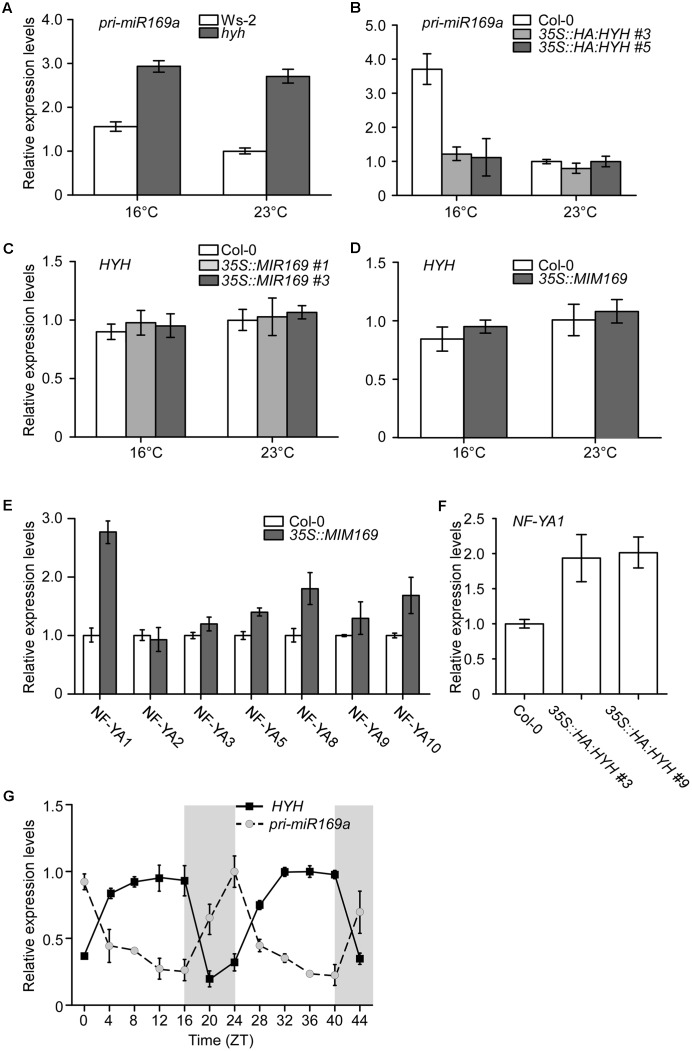
Measurement of *HYH* and *pri-miR169a* expression by qPCR. **(A,B)** The relative levels of *pri-miR169a* in Ws-2 and *hyh* mutants grown at 16 and 23°C **(A)**, and in Col-0 plants and two independent lines overexpressing *HYH-HA* **(B)**. **(C,D)** The relative levels of *HYH* transcripts in Col-0 and two independent lines overexpressing *MIR169a* **(C)** and *MIM169* **(D)** grown at 16 and 23°C. **(E)** Analysis of transcript levels of seven *Nuclear Factor Y Subunit A (NF-YA)* genes in Col-0 and plants overexpressing *MIM169*. **(F)** Analysis of *NF-YA* transcript levels in Col-0 and plants overexpressing *HYH-HA*. **(G)** Diurnal expression of *HYH* and *pri-miR169a* in wild-type plants. Gray boxes indicate the dark period. The results represent an average ± SD of three biological replicates.

To investigate the genetic relationship between *HYH* and *MIR169a*, *pri-miR169a* expression levels were measured using qPCR in *hyh* mutants grown at 16 and 23°C. The results showed that the levels of *pri-miR169a* were upregulated in *hyh* mutants at both temperatures when compared to the wild-type Ws plants (**Figure 7A**), indicating that *MIR169a* expression is induced upon the depletion of HYH. We also observed an increase of *pri-miR169a* transcript levels at 16°C in wild-type Ws-2 plants (**Figure 7A**), which is consistent with a previous report of higher accumulation of mature miR169a in wild-type Col-0 plants at 16°C (Lee et al., 2010).

We measured the levels of *pri-miR169a* transcripts in *35S::HA:HYH* plants to test whether the overexpression of *HYH* would negatively affect the expression of *pri-miR169a*. At 16°C, the levels of *pri-miR169a* transcripts in the *HYH*-overexpressing plants were suppressed by almost threefold as compared to that of wild-type plants (**Figure 7B**). Moreover, *pri-miR169a* levels were less responsive to the ambient temperature changes in *35S::HA:HYH* plants. At 23°C, the suppression of *pri-miR169a* by overexpression of *HYH* was not dramatic, probably because the *pri-miR169a* levels were already low at a high ambient temperature (23°C). To further investigate, we measured the levels of *HYH* transcripts in the *35S::MIR169a* plants and *35S:*:*MIM169* plants, in which the cleavage activity of most miR169 isoforms is inhibited (Todesco et al., 2010). Our results showed that the expression of *HYH* was unaltered by miR169a overexpression (**Figure 7C**), or inhibition of miR169 function (**Figure 7D**) at both temperatures, indicating that alteration of miR169 activity did not affect the transcript levels of *HYH*. This suggested that *HYH* did not act downstream of miR169a. The expression levels of *HYH* in Col-0 plants at both temperatures were similar, suggesting that the ambient temperature does not regulate *HYH* at the transcriptional level.

Mature miR169 consists of four isoforms produced from 14 loci (Jones-Rhoades and Bartel, 2004; German et al., 2008; Li et al., 2017), and each miR169 isoform targets specific *NF-YA* genes (Sorin et al., 2014). To identify the main target of miR169a, we analyzed the transcript levels of different *NF-YA*s. Only *NF-YA1* showed a major increase and was induced by more than 2.5-fold in *35S::MIM169* plants (**Figure 7E**), suggesting that *NF-YA1* might be the target gene of miR169a. However, as it is proposed that *35S::MIM169* plants block the action of three miR169 isoforms (i.e, miR169a, bc, and hijklmn, but not defg) (Todesco et al., 2010), the data do not rule out the possibility that *NF-YA1* might be the target of miR169bc and/or miR169hijklmn. Our results are in agreement with the notion that the depletion of mature miR169a is correlated with the increase of transcript levels of its target genes and *vice versa* (Lee et al., 2010). To evaluate whether *NF-YA1*, the target of miR169a, was positively regulated by the overexpression of *HYH*, the expression levels of *NF-YA1* were analyzed in *HYH*-overexpressing plants. The results showed that the levels of *NF-YA1* were indeed upregulated in *35S::HA:HYH* plants (**Figure 7F**). Since we observed a consistent relationship between expression of the negative regulator and the target gene of miR169, this result suggested that upregulation of *NF-YA1* was due to the depletion of miR169a by the overexpression of *HYH*.

We then analyzed the diurnal rhythm of *HYH* and *pri-miR169a* transcripts. We sampled 7- and 8-day-old wild-type Col-0 seedlings every 4 h from ZT0 to ZT44. Consistent with the previous report of induction of *HYH* expression by light (Li et al., 2017), *HYH* transcripts were consistently abundant during the light period and then decreased drastically in the dark until the next light period (**Figure 7G**). The levels of *pri-miR169a* showed an opposite pattern, remaining low during the light period, starting to increase at ZT16, and peaking at ZT24, just before the beginning of the next light period, when *HYH* transcription would be induced. Our results demonstrated a clear opposite relationship between the diurnal oscillation patterns of *HYH* and *pri-miR169a* transcripts, consistent with the negative role of HYH in the regulation of *MIR169a* transcription.

## Discussion

### Ambient Temperature Regulates *MIR169* at the Transcriptional Level via HYH

In this study, we characterized the promoter of *MIR169a*, an ambient temperature-responsive miRNA gene (Lee et al., 2010), and identified the *cis*-acting elements. We also identified a transcription factor that is responsible for the ambient temperature response. The 5′-promoter deletion GUS analyses revealed that the promoter region -1,505 to -1,007 bp upstream of *MIR169a* conferred ambient temperature-responsive expression (**Figure 3**). The DNA-affinity chromatography coupled with LC-MS showed that HYH bound to the -1,505/-1,007 promoter region (**Figure 4**). EMSA data showed that HYH protein directly bound to a derivative of G-box motif, which is positioned at -1,167/-1,157 in the promoter (**Figure 6D**). Mutation of the core G-box-like sequence prevented the interaction with HYH (**Figure 6D**), supporting the idea that the G-box-like motif is crucial for the interaction between HYH and *MIR169a*. The genetic interaction using *hyh* mutants and plants overexpressing *HYH*, *MIR169a*, and *MIM169* revealed that at a higher ambient temperature, HYH protein acted upstream of *MIR169a* as a negative regulator (**Figure 7**). Our finding on the negative role of HYH in the regulation of *MIR169a* expression is consistent with the observation that *hyh* mutants flower early (Holm et al., 2002) and overexpression of *MIR169* also produced an early flowering phenotype (Xu et al., 2014). We found that HYH protein levels were higher at 23°C than at 16°C (**Figure 6G** and Supplementary Figure 1B), whereas *HYH* transcript levels were not affected by ambient temperature changes (**Figure 7D**). This suggested that ambient temperature might regulate HYH levels post-transcriptionally. Interestingly, COP1, an E3 ubiquitin ligase that is known to promote degradation of HYH and HY5 (Holm et al., 2002), is more stable in the night and dawn at a low ambient temperature (Jang et al., 2015). This suggests that COP1 mediates HYH protein degradation at lower temperatures, thereby leading to lower DNA binding capacity at lower temperatures (**Figure 6F** and Supplementary Figure 1A). Nevertheless, since there are four *HYH* isoforms and all of them contain the conserved bZIP DNA-binding domain (Li et al., 2017), it would be interesting to investigate whether the different ambient temperatures favor different splicing variants and whether each protein variant has a different DNA-binding affinity.

**FIGURE 8:**
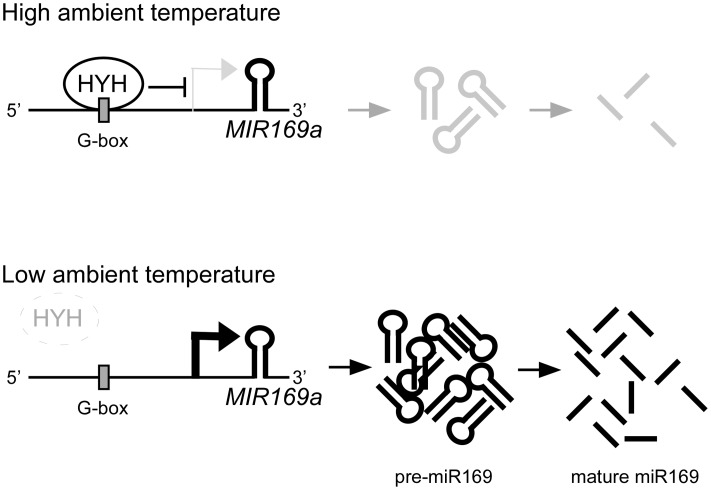
A proposed model for the function of HYH in regulating the expression of *MIR169a*. At higher ambient temperatures, HYH binds to the G-box-like motif in the promoter of *MIR169a* to repress its expression, which results in production of less pre-miR169a and less mature miR169. However, at lower ambient temperatures, HYH protein stability decreases and subsequently HYH enrichment on the G-box-like motif also decreases, and thus HYH fails to repress *MIR169a* expression, which results in production of more pre-miR169a and mature miR169. Other environmental factors such as light, or biotic and abiotic stresses may also affect the transcriptional regulation of *MIR169a*.

Based on our findings, we suggest a working model (**Figure 8**) to demonstrate the potential upstream negative regulation of *MIR169a* by ambient temperature via HYH protein. Upon exposure to a higher ambient temperature, HYH binds to the G-box-like motif in the promoter of *MIR169a* to repress its expression and subsequently decrease the abundance of miR169 at higher temperatures. At a lower temperature, HYH fails to bind to the *MIR169a* promoter; thus, *MIR169a* transcription occurs, which leads to more pre-miR169a and mature miR169a production. Since HYH is known to be involved in photomorphogenesis and light signaling (Oyama et al., 1997; Li et al., 2017), our results also suggest the existence of crosstalk between the ambient temperature and light signaling mechanisms.

### A Derivative of the G-Box Motif as a Novel Ambient Temperature-Responsive Element

The expression of *MIR169a* is affected by ambient temperature, making its promoter a model for enhancing our understanding of the transcriptional mechanism controlled by ambient temperature. In this study, we characterized an ambient temperature-responsive promoter in transgenic Arabidopsis plants. Our findings suggest that a G-box-like DNA motif at -1,167/-1,157 in the promoter of *MIR169a* could confer ambient temperature-responsive *GUS* expression (**Figures 3**, **4**). The G-box motif is a *cis*-acting element that regulates responses to UV irradiation, anaerobiosis, abscisic acid, and light (Giuliano et al., 1988). Several bZIP transcription factors have high G-box-binding affinity (Vinson et al., 1989; Ellenberger et al., 1992). Since the deletion of the promoter region containing the G-box-like motif attenuated the ambient temperature response of the *MIR169a* promoter (**Figures 3**, **4**), and the mutation of the core motif failed to interact with HYH protein (**Figure 6D**), we conclude that the G-box-like motif is required for the ambient temperature response of the *MIR169a* promoter.

Previously identified *cis*-acting elements involved in high and low temperature perception mechanisms include Abscisic Acid Response Element (ABRE), C-repeat/DRE (Yamaguchi-Shinozaki and Shinozaki, 1994; Stockinger et al., 1997), CCAAT (Sahara et al., 1999), Low Temperature Response Element (LTRE) (Jiang et al., 1996), Stress Response Element (STRE), SAR (Haralampidis et al., 2002), and Heat Shock Element (HSE) (Schoffl et al., 1998). The G-box-like motif (5′-CACGCAAAAG-3′) identified in this study did not show sequence similarity to any known temperature-responsive elements, suggesting that it is a novel *cis*-acting element conferring ambient temperature responsiveness. Considering that the G-box is a hexameric sequence (5′-CACGTG-3′), a derivative of the G-box identified in our study, despite its sequence similarity to the G-box, could possibly be an entirely different type of *cis*-acting element. The sequences flanking the core G-box affect the specificity of protein binding (Williams et al., 1992); therefore, a detailed mutational analysis of the core sequence must be performed to elucidate, define, and classify the type of *cis*-acting element we identified here. The G-box motif and its binding proteins HY5 and HYH also act in photomorphogenesis and light-sensing mechanisms (Holm et al., 2002); therefore, our findings additionally suggest their role in ambient temperature signaling and possible crosstalk between the two mechanisms.

### Crosstalk between Ambient Temperature Sensing and Light-Sensing Mechanisms

Temperature and light are environmental cues recognized by plants as indicators of seasonal changes. Therefore, the mechanisms that perceive and integrate temperature and light signals are crucial for plant survival. There is an increasing body of evidence suggesting the existence of crosstalk between the ambient temperature and light-sensing mechanism in Arabidopsis; for instance, emerging evidence shows that phytochromes act as temperature sensors (Jung et al., 2016; Legris et al., 2016). Previous studies reported that a group of bHLH transcription factors, the phytochrome interacting factors (PIFs), regulate the light-responsive transcriptional network (Leivar and Quail, 2011) by interacting with the photoreceptors phyA and phyB (Khanna et al., 2004). PIFs and the bZIP transcription factor HY5, a close homolog of HYH, are antagonists that both target a G-box DNA motif; they form an activation-suppression transcriptional module responsive to light and temperature signals (Toledo-Ortiz et al., 2014). As the closest homolog of HY5, HYH can also bind to G-box motifs. HY5 and HYH show functional redundancy and are subjected to COP1-mediated dark-specific degradation as they contain the conserved COP1-binding domain (Holm et al., 2002). More recent studies further demonstrated that temperature and light sensing mechanisms share common signaling components during signal transduction. Delker et al. (2014) demonstrated overlapping elements of the two pathways in the DET1-COP1-HY5 module. Although the function of HYH has not been as well characterized, it has been shown that HYH also participates in the light-signaling mechanism, unlike its homolog HY5 (Singh et al., 2012). As *HYH* expression was previously shown to be induced by light (Li et al., 2017), our findings additionally suggest that it also exhibits a diurnal oscillation, being downregulated in the dark period (**Figure 7G**). Moreover, the opposite diurnal shifts observed in *MIR169a* and *HYH* expression levels either resulted from HYH depletion (**Figure 7G**) or light signals that might directly regulate *MIR169a* expression due to the presence of multiple light-responsive *cis*-acting elements in its promoter (**Figure 2A**). These observations support the notion that the ambient temperature regulation of *MIR169a* expression via the HYH might be modulated by crosstalk with the light-sensing mechanism.

Understanding how plants response to fluctuating temperature at the molecular level is a crucial step that must be taken to predict plant responses to climate change, mitigate the damage to agriculture, and support the conservation of biodiversity. Without activating temperature stress responses, ambient temperature influences plant growth and development (Samach and Wigge, 2005), disease resistance (Yang and Hua, 2004; Wang et al., 2009), and the circadian clock (Boikoglou et al., 2011). However, the elucidation of the ambient temperature sensing mechanism has been a challenge, considering that plants in their natural habitats are exposed to multiple environmental factors, and temperature signals are perceived in combination with other environmental stimuli. To understand how plants process complex environmental signals, on top of studies in environmentally controlled conditions, further investigation on the molecular interaction of plants in their natural habitats will give us a better insight into how plants perceive and response to ambient temperature changes.

## Author Contributions

PS designed, performed, and analyzed data from the experiments. HS performed the experiments and data analysis. JA designed and supervised the study. PS, HS, and JA wrote the manuscript.

## Conflict of Interest Statement

The authors declare that the research was conducted in the absence of any commercial or financial relationships that could be construed as a potential conflict of interest.

## Footnotes

1 https://blast.ncbi.nlm.nih.gov/Blast.cgi?PAGE=Proteins

2 http://bioinformatics.psb.ugent.be/webtools/plantcare/html/
