# Supplementary material for: Elongated Hypocotyl 5-Homolog (HYH) Negatively Regulates Expression of the Ambient Temperature-Responsive MicroRNA Gene MIR169
Source: Front Plant Sci. 2017 Dec 7;8:2087. doi: 10.3389/fpls.2017.02087 (PMC5725467; doi:10.3389/fpls.2017.02087)
Supplement: Supplementary file 1 [file Table_1.DOCX]

**Supplementary Table 1 | Oligonucleotide sequences used for cloning, RT-PCR, qPCR, EMSA, and ChIP-qPCR in this study.**

| **Gene** | **Primer** | **Purpose** | **Sequence (5′ to 3′)^*^** | **Direction** |
| --- | --- | --- | --- | --- |
| pri-MIR169a | JH7266 | Absolute qPCR, qPCR | AAAGTAACATGATCGGCAAGTTGTCC | Sense |
|  | JH7267 | Absolute qPCR, qPCR | GCGACACAAAGTAACGTGTAGCC | Anti-sense |
|  | JH11863 | Cloning | gactaggatccGTGACGAAAGTAGTGTGCAG | Sense |
|  | JH11864 | Cloning | gactagtcgacGCGACACAAAGTAACGTGTAG | Anti-sense |
| pri-MIR169b | JH7268 | Absolute qPCR | CCCAACGGAGTAGAATTGCATGAAGTG | Sense |
|  | JH7269 | Absolute qPCR | GCCGAAGGACAACTTGCCAGAAATATG | Anti-sense |
| pri-MIR169c | JH7270 | Absolute qPCR | TGTGTGTTGGGGCAAGAAGTGTAAC | Sense |
|  | JH7271 | Absolute qPCR | GCCTAGAATGAGAGGAAGAGACAGTTG | Anti-sense |
| pri-MIR169d | JH7272 | Absolute qPCR | CAAGGATGACTTGCCGATGTTATCAAC | Sense |
|  | JH7273 | Absolute qPCR | AAAGAAGGAAACAGAGCCAAGGTCAAC | Anti-sense |
| pri-MIR169e | JH7274 | Absolute qPCR | AGGATGACTTGCCGATTTTCTCAACG | Sense |
|  | JH7275 | Absolute qPCR | AAAGAAGGGAAGGAAACAGAGCCAAAG | Anti-sense |
| pri-MIR169f | JH7276 | Absolute qPCR | AACGAATGGAATTGAGCCAAGGATGAC | Sense |
|  | JH7277 | Absolute qPCR | AGAATAGAGAACGAAGCAGAGCCAAGG | Anti-sense |
| pri-MIR169g | JH7278 | Absolute qPCR | GCATGGAAGAATAGAGAATGAGG | Sense |
|  | JH7279 | Absolute qPCR | GCCGGACACCAGAATCAGTT | Anti-sense |
| pri-MIR169h | JH7280 | Absolute qPCR | GGATGACTTGCCTGCGTTTTAGACC | Sense |
|  | JH7281 | Absolute qPCR | ACTGCCTGACGACCAACCAACTC | Anti-sense |
| pri-MIR169i | JH7282 | Absolute qPCR | AAGGATGACTTGCCTGACTCTTTGTG | Sense |
|  | JH7283 | Absolute qPCR | CATATAAGGATAGCCAAGGAGACTGCC | Anti-sense |
| pri-MIR169j | JH7284 | Absolute qPCR | TAGTAGCCAAGGATGACTTGCCTGATC | Sense |
|  | JH7285 | Absolute qPCR | AGCCAAGGAGACTGCCTGAAACC | Anti-sense |
| pri-MIR169k | JH7286 | Absolute qPCR | ACTTGCCTGCTTCTCTGAACAAAATGG | Sense |
|  | JH7287 | Absolute qPCR | GGATAGCCAAGGAGACTGCCTGATG | Anti-sense |
| pri-MIR169l | JH7288 | Absolute qPCR | AATAGCCAAGGATGACTTGCCTGATC | Sense |
|  | JH7289 | Absolute qPCR | AGAGACTGCCTGAAACCTAACCCG | Anti-sense |
| pri-MIR169m | JH7290 | Absolute qPCR | GTAGCCAAGGATGACTTGCCTGTTTC | Sense |
|  | JH7291 | Absolute qPCR | GGATAGCCAAGGAGACTGCCTGATG | Anti-sense |
| pri-MIR169n | JH7292 | Absolute qPCR | AGAGAGGTCTAACATGGCGGAAAGC | Sense |
|  | JH7293 | Absolute qPCR | AGATCAGGCAAGTCATCCTTGGCTAC | Anti-sense |
| pMIR169.1 | JH10674 | Cloning | gcatgcAAACGTGGTTCACAAAAACC | Sense |
| pMIR169.2 | JH10675 | Cloning | gcatgcTCATATCCAACCATAAACGG | Sense |
| pMIR169.3 | JH10676 | Cloning | gcatgcATGATAGATTAGGTGCCTAC | Sense |
| pMIR169.4 | JH10677 | Cloning | gcatgcGGCAAACGGGTTCCTAATC | Sense |
| pMIR169.5 | JH10678 | Cloning | gcatgcGACAAAAACCCTAGATTTGATC | Sense |
| pMIR169-R | JH10679 | Cloning | tctagaGTTTCTTTGCGTTTCTCTTG | Anti-sense |
| *HYH.2* | JH11935 | Absolute qPCR | GAAAAGATGCAATTTCCTTTGGTTTG | Sense |
|  | JH11936 | Absolute qPCR | TTCTTGTGGGAAGAAGACGAACTC | Anti-sense |
|  | JH11859 | Cloning | gactaggatccATGTCTCTCCAACGACCCAATG | Sense |
|  | JH11860 | Cloning | gactagtcgacTTAGTGATTGTCATCAGTTTTAGGCC | Anti-sense |
| *HYH.3* | JH11937 | Absolute qPCR | GCACAAAACTGGTAGGTGTTGAAAG | Sense |
|  | JH11938 | Absolute qPCR | GTTGATCCAGCTGCTTCCATGTC | Anti-sense |
| *NF-YA1* | JH12429 | qPCR | GGCTGTCGGGTTTTCTGAATCG | Sense |
|  | JH12430 | qPCR | TGCATCTCAGTGGAAATTCACAGCA | Anti-sense |
| *NF-YA2* | JH12431 | qPCR | ATCTGTCGGAGAGACAGGACAACG | Sense |
|  | JH12432 | qPCR | TCCAAGTTCCAAGCAAGGTGATTGC | Anti-sense |
| *NF-YA3* | JH12518 | qPCR | TTGCTCCTGCACAGGCTAATTTCTC | Sense |
|  | JH12519 | qPCR | AGCTCTGCTGGTAAAGGAACACGAC | Anti-sense |
| *NF-YA5* | JH12433 | qPCR | TGGCTCCTGAATATGCATCAACACC | Sense |
|  | JH12434 | qPCR | TGCGACGGAGAATCGCATGATAC | Anti-sense |
| *NF-YA8* | JH12520 | qPCR | TAATGCCTGCGGCTTACCTACCACA | Sense |
|  | JH12521 | qPCR | TGCATGGAATTGCTTTGCATTGAC | Anti-sense |
| *NF-YA9* | JH12522 | qPCR | TGATGGGAGCCTATGGACATCATCC | Sense |
|  | JH12523 | qPCR | CGTGAATGCTAAACAGTACCAGGCG | Anti-sense |
| *NF-YA10* | JH12435 | qPCR | CCCGAGCAAAGGCTGAAAAACTGAG | Sense |
|  | JH12436 | qPCR | CGCATCAGCTGTCTTGGTGTTCAAG | Anti-sense |
| miR169a A-box | JH12128 | EMSA | AGCTTTCATACGTAAAATCTCT | Sense |
|  | JH12129 | EMSA | AGAGATTTTACGTATGAAAGCT | Anti-sense |
| miR169a GATA-box 1 | JH12130 | EMSA | CTTATTTTAATTGATAAATA | Sense |
|  | JH12131 | EMSA | TATTAAAATATCAATTTAAG | Anti-sense |
| miR169a GATA-box 2 | JH12132 | EMSA | ATCTTTTCGATAACTTTGAG | Sense |
|  | JH12133 | EMSA | CTCAAAGTTATCGAAAAGAT | Anti-sense |
| miR169a G-box | JH12134 | EMSA | TGTACGGCCACGCAAAAGTGACCTAC | Sense |
|  | JH12135 | EMSA | GTAGGTCACTTTTGCGTGGCCGTACA | Anti-sense |
| miR169a NC1 | JH12136 | EMSA | ATCTTCTGTTTAACAACATATG | Sense |
|  | JH12137 | EMSA | CATATGTTGTTAAACAGAAGAT | Anti-sense |
| miR169a mG-box | JH12293 | EMSA | TGTACGGCACATACCCCTTGACCTAC | Sense |
|  | JH12294 | EMSA | GTAGGTCACTTTTGCGTGGCCGTACA | Anti-sense |
| miR169a P1 | JH13154 | ChIP | gaaaaactataaaaatgtacggcc | Sense |
|  | JH13155 | ChIP | ctcaaagttatcgaaaagatcaaa | Anti-sense |
| miR169a NC2 | JH13160 | ChIP | ggtgaaaagagacggtcg | Sense |
|  | JH13161 | ChIP | catccaagtaaccctagtccc | Anti-sense |
