# Supplementary material for: Elongated Hypocotyl 5-Homolog (HYH) Negatively Regulates Expression of the Ambient Temperature-Responsive MicroRNA Gene MIR169
Source: Front Plant Sci. 2017 Dec 7;8:2087. doi: 10.3389/fpls.2017.02087 (PMC5725467; doi:10.3389/fpls.2017.02087)
Supplement: Supplementary file 2 [file Image_1.PDF]

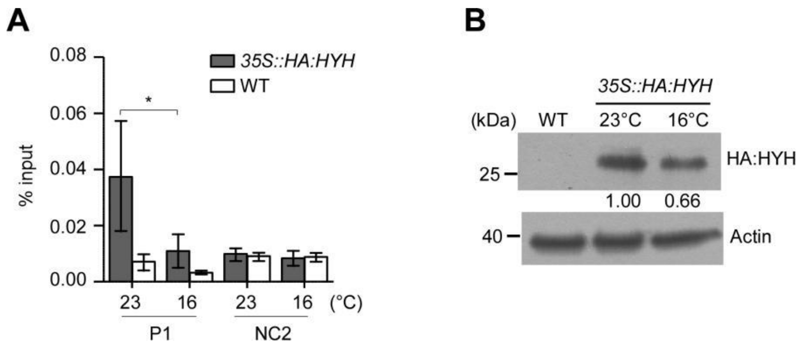

Supplementary Figure 1. | Results of the biological replicates that are not presented in the main text

(A) Second biological replicate of ChIP-qPCR assay shown in Figure 6F. All values are presented as the mean ± SD of three technical replicates, $*p \leq 0.05$ (Student's t-test).
(B) Second biological replicate of western blot data shown in Figure 6G. Bands were quantified using Image J software and the numbers below each band denote fold change relative to the HA:HYH level at 23°C.
